# Genomic Study of High-Risk Clones of *Enterobacter hormaechei* Collected from Tertiary Hospitals in the United Arab Emirates

**DOI:** 10.3390/antibiotics13070592

**Published:** 2024-06-26

**Authors:** Akela Ghazawi, Febin Anes, Shaimaa Mouftah, Mohammed Elbediwi, Awase Baig, Muna Alketbi, Fatema Almazrouei, Mariam Alhashmi, Norah Alzarooni, Ashrat Manzoor, Ihab Habib, Nikolaos Strepis, Anju Nabi, Mushtaq Khan

**Affiliations:** 1 Department of Microbiology and Immunology, College of Medicine and Health Sciences, United Arab Emirates University, Al Ain P.O. Box 15551, United Arab Emirates; akelag@uaeu.ac.ae (A.G.); 202070161@uaeu.ac.ae (A.B.); 201905897@uaeu.ac.ae (M.A.); 202007513@uaeu.ac.ae (F.A.); 202003630@uaeu.ac.ae (M.A.); 202006258@uaeu.ac.ae (N.A.); 700043295@uaeu.ac.ae (A.M.); 2 Veterinary Public Health Research Laboratory, Department of Veterinary Medicine, College of Agriculture and Veterinary Medicine, United Arab Emirates University, Al Ain P.O. Box 15551, United Arab Emirates; hfebin@uaeu.ac.ae (F.A.); i.habib@uaeu.ac.ae (I.H.); 3 Department of Biomedical Sciences Program, University of Science and Technology, Zewail City of Science and Technology, Giza 12578, Egypt; 201590053@uaeu.ac.ae; 4 Evolutionary Biology, Institute for Biology, Freie Universität Berlin, 14163 Berlin, Germany; mohammed.elbediwi@fu-berlin.de; 5 Animal Health Research Institute, Agriculture Research Centre, Cairo 12618, Egypt; 6 Department of Medical Microbiology & Infectious Diseases, Erasmus University Medical Centre (Erasmus MC), 3015 GD Rotterdam, The Netherlands; n.strepis@erasmusmc.nl; 7 Microbiology and Immunology Department, Dubai Hospital, Dubai P.O. Box 53735, United Arab Emirates; anabi@dubaihealth.ae

**Keywords:** *Enterobacter hormachei*, MDR, whole-genome sequencing, plasmids, incompatibility types

## Abstract

*Enterobacter hormaechei* has emerged as a significant pathogen within healthcare settings due to its ability to develop multidrug resistance (MDR) and survive in hospital environments. This study presents a genome-based analysis of carbapenem-resistant *Enterobacter hormaechei* isolates from two major hospitals in the United Arab Emirates. Eight isolates were subjected to whole-genome sequencing (WGS), revealing extensive resistance profiles including the *bla*_NDM-1_, *bla*_OXA-48_, and *bla*_VIM-4_ genes. Notably, one isolate belonging to ST171 harbored dual carbapenemase genes, while five isolates exhibited colistin resistance without *mcr* genes. The presence of the type VI secretion system (T6SS), various adhesins, and virulence genes contributes to the virulence and competitive advantage of the pathogen. Additionally, our isolates (87.5%) possessed ampC β-lactamase genes, predominantly *bla*_ACT_ genes. The genomic context of *bla*_NDM-1_, surrounded by other resistance genes and mobile genetic elements, highlights the role of horizontal gene transfer (HGT) in the spread of resistance. Our findings highlight the need for rigorous surveillance, strategic antibiotic stewardship, and hospital-based WGS to manage and mitigate the spread of these highly resistant and virulent pathogens. Accurate identification and monitoring of *Enterobacter cloacae* complex (ECC) species and their resistance mechanisms are crucial for effective infection control and treatment strategies.

## 1. Introduction

The genus *Enterobacter*, a member of the notorious ESKAPE pathogens (*Enterococcus faecium*, *Staphylococcus aureus*, *Klebsiella pneumoniae*, *Acinetobacter baumannii*, *Pseudomonas aeruginosa*, and *Enterobacter* species), represents a global threat to human health [1]. These bacteria are particularly worrisome because they can develop resistance to multiple antibiotics, including those considered a last resort [2]. Among *Enterobacter* species, the *Enterobacter cloacae* complex (ECC) has developed specific mechanisms of resistance to carbapenems and other β-lactam antibiotics [2,3,4]. Consequently, ECC species are capable of causing various infections, including bloodstream, intra-abdominal, urinary tract, gastrointestinal, and pulmonary infections, which can culminate in life-threatening bacteremia, with a mortality rate of up to 40% [5,6,7]. The *Enterobacter cloacae* complex (ECC) is often linked with the multidrug-resistant (MDR) phenotype due to its substantial ability to acquire genetic elements that encode resistance genes. Additionally, ECC is inherently resistant to multiple antibiotics, such as ampicillin, amoxicillin/clavulanic acid, cephamycin, and first- and second-generation cephalosporins, due to the chromosomal encoding of AmpC-type β-lactamase. More concerning is the emergence of the carbapenem-resistant *Enterobacter cloacae* complex (CR-ECC), which has created significant challenges for the clinical treatment of infections [8].

At present, *Enterobacter hormaechei* has emerged as one of the most prevalent pathogens among CR-ECC isolates in hospital settings, with its infection rate rising annually and several outbreaks having been reported in recent years. Notably, various carbapenemases, such as New Delhi metallo-β-lactamase-1 (*NDM-1*), *Klebsiella pneumoniae* carbapenemase-2 (*KPC-2*), and German-Imipenemase-1 (*GIM-1*), have been identified in *Enterobacter hormaechei*. Typically, colistin is one of the last-resort antibiotics for treating infections caused by CR-ECC isolates; however, the global spread of plasmid-mediated colistin resistance genes (*mcr*) has challenged its efficacy. Besides colistin, tigecycline might be the only remaining treatment option for CR-ECC infections. Nevertheless, since its approval in 2005, tigecycline-resistant strains have increasingly been found in clinical settings. Recent studies have shown that *Enterobacter hormaechei* has developed resistance to tigecycline through the acquisition of plasmid-borne *tet(A)* variants and *tet(X4)* [9]. 

Accurate identification of *Enterobacter* species poses significant challenges for clinicians and researchers. Although automated microbial identification systems and mass spectrometry have proven reliable in identifying many bacterial species, they often lack the precision required to distinguish *Enterobacter* species [1]. Therefore, clinical laboratories often classify *Enterobacter* species under the umbrella of the ECC, making it difficult for them to establish a direct correlation between antimicrobial resistance and specific *Enterobacter* species [1].

There has been a significant increase in research on the detection of carbapenemase-encoding genes in *Enterobacter* strains. A recent study by Zong et al. examined 4899 high-quality *Enterobacter* genome sequences from GenBank with the dual goals of precise species identification and screening of resistance genes [7]. The results showed that *Enterobacter hormaechei* subsp. *xiangfangensis* is the most widespread species worldwide, with the *bla*_NDM_ gene predominantly occurring in China. Several high-risk *Enterobacter* clones associated with carbapenem resistance have also been discovered. In particular, the carbapenemase-producing ST171 *Enterobacter hormaechei* subsp. *xiangfangensis* has been identified as a high-risk lineage in the United States [10,11,12]. Additionally, globally distributed clones such as ST90, ST93, and ST114 from *Enterobacter hormaechei* subsp. *xiangfangensis* and ST78 from *Enterobacter hoffmannii* have been associated with several carbapenemases (VIM, NDM, KPC, and OXA-48) [13]. Based on a comprehensive literature search and to the best of our knowledge, there are no published studies specifically focusing on *Enterobacter hormaechei* in the UAE or the broader Gulf region. While there is substantial research on carbapenem-resistant *Enterobacteriaceae* (CRE) in the UAE and neighboring countries such as Saudi Arabia, Qatar, and Oman, these studies have generally addressed broader categories of CRE without specifically highlighting *Enterobacter hormaechei*. This gap in the literature highlights the importance of our current study, which provides novel insights into the genetic characteristics and resistance mechanisms of *Enterobacter hormaechei* isolates in the UAE.

The purpose of this study was to characterize carbapenem-resistant *Enterobacter hormaechei* isolates from the UAE, analyze their antimicrobial resistance genes, and elucidate their epidemiological and phylogenetic characteristics using whole-genome sequencing (WGS). This research aimed to provide a comprehensive understanding of this specific pathogen in the UAE, contributing valuable insights to the global fight against antibiotic-resistant bacteria. For this study, we collected and analyzed clinical samples of *Enterobacter hormaechei* from two healthcare facilities in the Abu Dhabi Emirate, UAE. Using advanced genomic techniques, we performed WGS to identify resistance genes, determine phylogenetic relationships, and track the epidemiology of these strains. This is the first study to provide such an in-depth analysis of carbapenem-resistant *Enterobacter hormaechei* in the UAE, marking a significant step toward understanding and managing this health threat in the region.

## 2. Results

### 2.1. Genome-Based Species and ST Identification of Enterobacter spp.

Eight isolates were identified as *Enterobacter hormaechei*, with two belonging to the *steigerwaltii* subspecies and one to the *xiangfangensis* subspecies (Table 1). The bacterial isolates were derived from different sites on the bodies of the patients, including wound swabs, sputum, tissue, and urine. Of the sequence types (STs) identified, ST90 was the predominant one at 37.5% (3/8), followed by ST124 at 25% (2/8). Additionally, three different singleton STs were observed: ST182, ST269, and ST171, each occurring once (n = 3; Table 1).

### 2.2. Antimicrobial Susceptibility Profile of Enterobacter hormaechei

The minimum inhibitory concentration (MIC) results are presented in Table 2, which indicate that all eight isolates exhibit a broad spectrum of drug resistance, classifying them as multidrug-resistant bacteria. Specifically, all eight *Enterobacter* isolates were resistant to piperacillin/tazobactam, cefotaxime, ceftazidime, ceftolozane/tazobactam, imipenem, and gentamicin. Furthermore, the majority of the isolates (seven out of eight) showed phenotypic resistance to CAZ/AVI, cefepime, meropenem, and ciprofloxacin. Of these isolates, five were resistant to trimethoprim/sulfamethoxazole, and only one showed resistance to tigecycline. Variable susceptibility to amikacin was observed, with none of the isolates being resistant. Notably, five isolates—three of which belonged to ST90 (CRE48, CRE52, and CRE60) and the remaining two to ST-124 (CRE41 and CRE46)—exhibited resistance to colistin.

### 2.3. Antimicrobial Resistance Genes

**Figure 1:**
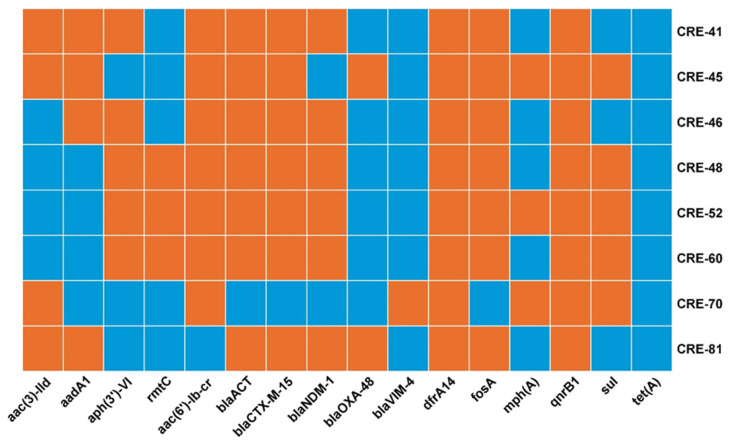
Antimicrobial resistance genes and resistome profile of *Enterobacter hormaechei* isolates. Orange tiles indicate the presence of the gene, blue indicates the absence of the gene.

All isolates exhibited a broad range of antimicrobial resistance (AMR) genes, which confer resistance to different classes of antimicrobial agents, including carbapenems, β-lactams, sulfonamides, aminoglycosides, fosfomycin, and quinolones (as shown in Figure 1). 

Macrolide resistance genes were detected in only two isolates. One contained the *mph(A)* gene, while the other carried both *mph(A)* and *ere(A)* genes. Regarding carbapenemases, a single isolate of the *xiangfangensis* subspecies (ST171-CRE81) showed dual carbapenemase production involving *bla*_NDM-1_ and *bla*_OXA-48_. Furthermore, ST269 (CRE70) was associated with *bla*_VIM-4_. Moreover, all three ST90 (CRE48, CRE52, and CRE60) isolates carried *bla*_NDM-1_ and both ST124 (CRE41 and CRE46) isolates possessed this gene. ST182 (CRE45) was identified as a carrier of *bla*_OXA-48_. For aminoglycoside resistance, the rmtC gene was only present in isolates with ST90 (CRE48, CRE52, and CRE60). Regarding extended-spectrum β-lactamases (ESBLs), all isolates carried *bla*_CTX-M-15_, except for CRE70. All isolates were found to lack the *mcr* gene. Additionally, 87.5% of the isolates carried AmpC-B-lactamase genes; predominantly *bla*_ACT_ genes. Among these *Enterobacter hormaechei* isolates, *bla*_ACT-7_, *bla*_ACT-15_, and *bla*_ACT-16_ were identified. A detailed resistome profile is provided in Figure 1.

### 2.4. Virulence Genes

Each of the isolates exhibited distinct virulence determinants, as shown in Table 3. Notably, invasion determinants (*ibeB*, *cheB*, *cheW*, *cheY*, *cheR*, and *motA*) were absent in ST82 (CRE45), ST269 (CRE70), and ST171 (CRE81). The immune evasion mechanisms (*gale* and *gtrA*) were present only in ST182 (CRE45) and ST171 (CRE81). Additionally, ST90 (CRE48, CRE52, and CRE60) and ST269 (CRE70) significantly lacked biofilm formation determinants. However, the secretion system was a common feature present in all isolates analyzed. Regarding adherence, type 3 fimbriae were present only in ST124 (CRE41 and CRE46). The secretion system (T6SS) was present in all isolates.

### 2.5. Plasmids and Genetic Environment of Carbapenemase Genes

In our genome analysis and plasmid typing of *Enterobacter hormaechei* strains, we observed a notable trend: the IncFII and IncFIB plasmids were consistently present in all isolates, except for CRE70 and CRE81, which lacked the IncFIB plasmid. This pattern highlights the potential role of these plasmids in the behavior or antibiotic resistance profile of the bacteria. Further delving into specific sequence types, we found that ST171 (CRE81) and ST182 (CRE45) isolates harbored the IncL plasmid, whereas the ST269 (CRE70) isolate was characterized by the presence of IncC, IncHI1A, and IncN plasmids.

**Figure 2:**
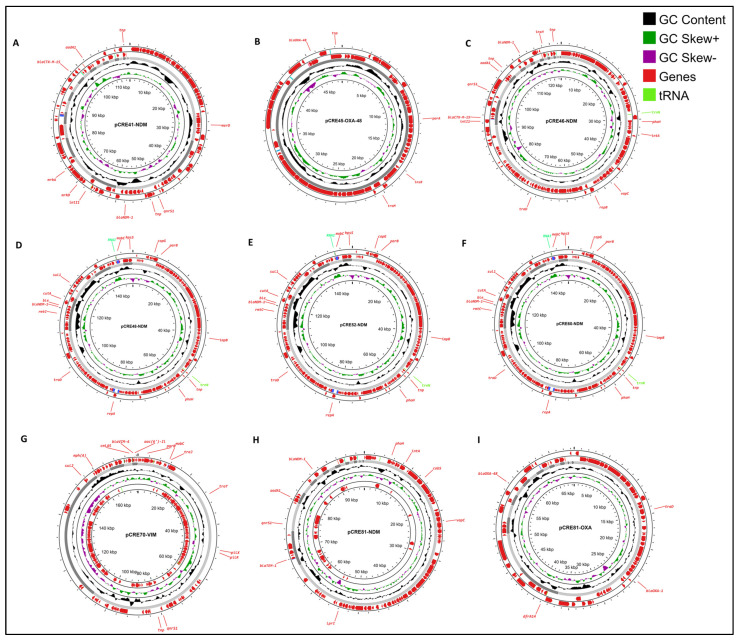
Circular maps of plasmids isolated from *Enterobacter hormaechei* isolates. This figure represents the circular maps of plasmids pCRE41-NDM (**A**), pCRE45-OXA-48 (**B**), pCRE46-NDM (**C**), pCRE48-NDM (**D**), pCRE52-NDM (**E**), pCRE60-NDM (**F**), pCRE70-VIM (**G**), pCRE81-NDM (**H**), and pCRE81-OXA (**I**). Annotations include GC content (black), GC Skew+ (green), GC Skew- (purple), Genes (red), and tRNA (light green). The plasmids contain several resistance genes: pCRE41-NDM (A) includes *bla*_NDM1_, *bla*_CTX-M-15_, *qnrS1*, and *aadA1*; pCRE45-OXA-48 (B) includes *bla*_OXA-48_; pCRE46-NDM (C) includes *bla*_NDM1_, *bla*_CTX-M-15_, *qnrS1*, and *aadA1*; pCRE48-NDM (D), pCRE52-NDM €, and pCRE60-NDM (F) include *bla*_NDM1_, *rmtC*, *sul1*, and *dfrA14*; pCRE70-VIM (G) includes *bla*_VIM-4_, *mphA*, *sul2*, *cmlA5*, and *aac(6’)-II*; pCRE81-NDM (H) includes *bla*_NDM-1_, *bla*_TEM-1_, *qnrS1*, and *aadA1*; and pCRE81-OXA (I) includes *bla*_OXA-48_, *bla*_OXA-1_, and *dfrA14*. The outermost ring shows the annotated genes, while the inner rings display GC content and GC skew.

A correlation emerged between certain carbapenemase genes and specific plasmid types, as detailed in Table 4 and Figure 2. For instance, the *bla*_NDM-1_ gene was predominantly associated with IncFII conjugative plasmids. These plasmids varied in size and harbored additional resistance genes in various combinations, as observed in isolates CRE41, CRE46, CRE48, CRE52, CRE60, and CRE81. In the case of *bla*_OXA-48_, we identified its location on an IncL/M plasmid in the ST182 isolate (CRE45) and ST171 isolate (CRE81). The *bla*_VIM-4_ gene was associated with IncC (CRE70).

**Figure 3:**
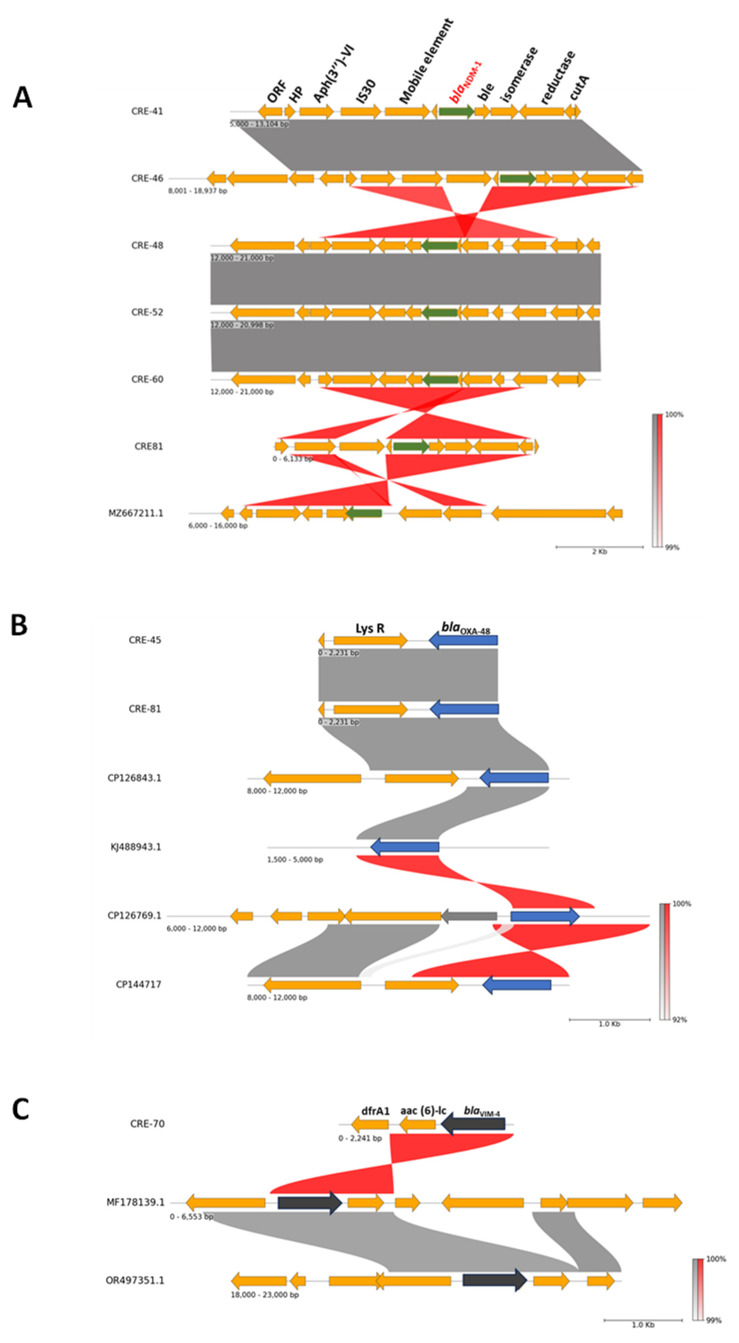
Genetic context and comparative analysis of resistance genes in *Enterobacter hormaechei* isolates. (**A**) Genomic context of the *bla*_NDM-1_ gene. The *bla*_NDM-1_ gene is surrounded by a cluster of genes and mobile genetic elements that may contribute to its spread and stability, including *aph(3′)-VI* (associated with aminoglycoside resistance), IS30 (an insertion sequence), and ble (encoding a bleomycin resistance protein), as well as putative genes for isomerase and reductase. (**B**) Genetic context of the *bla*_OXA-48_ gene. The *bla*_OXA-48_ gene is consistently associated with the presence of a transcriptional regulator (LysR). (**C**) Genetic context of the *bla*_VIM-4_ gene. The *bla*_VIM-4_ gene co-locates with the aminoglycoside resistance gene *aac(6′)-lc* and the trimethoprim resistance gene *dfrA1*.

Our results identified the genomic context of *bla*_NDM-1_. This gene is surrounded by several other genes and mobile genetic elements, which were identified consistently across multiple isolates and may contribute to the spread and stability of *bla*_NDM-1_. In particular, genes such as *aph(3′)-VI*, which is associated with aminoglycoside resistance, and other elements such as IS30 (an insertion sequence), as well as putative genes encoding a bleomycin resistance protein (ble), an isomerase, and a reductase, are part of this genomic landscape. These genes and elements are shown as arrows pointing in the direction of transcription, with *bla*_NDM-1_ clearly in-between, in Figure 3A.

The isolates exhibit varying degrees of genetic similarity to one another, as indicated by the red shading connecting them. In particular, the connections between the *bla*_NDM-1_ gene in isolates CRE46 and CRE48, as well as between CRE52 and CRE60, are characterized by intense red bands, indicating a high level of sequence similarity, close to 100%. This implies that the *bla*_NDM-1_ gene has been conserved with little variation, possibly indicating a more recent and common origin of these gene segments among the isolates. The extensive gray shading suggests large regions of shared genomic content, while the red lines indicate a strong genetic relationship specifically linked to the *bla*_NDM-1_ gene, supporting the hypothesis of horizontal gene transfer events that could spread resistance mechanisms across different bacterial hosts.

In summary, the detailed genetic context of the *bla*_NDM-1_ gene presents a conserved cluster of genes among isolates CRE41, CRE46, CRE48, CRE52, CRE60, CRE81, and MZ667211.1, which are associated with antibiotic resistance. The high sequence similarity among these isolates, particularly between the *bla*_NDM-1_ gene regions, highlights the potential for cross-transmission and the persistence of this resistance gene within different bacterial populations.

Additionally, the analysis highlighted similarities among isolates harboring the *bla*_OXA-48_ gene, where it is consistently associated with the presence of a transcriptional regulator (LysR), as depicted in Figure 3B. Regarding *bla*_VIM-4_, the genetic comparison revealed that this gene in CRE70 co-locates with the aminoglycoside resistance gene (*aac(6′)-lc*), a pattern also observed in other isolates. Furthermore, this genetic cassette includes an additional resistance gene encoding for trimethoprim resistance (*dfrA1*), underscoring the complexity of the resistance mechanisms in this particular isolate (Figure 3C).

### 2.6. Phylogenetic Tree of Enterobacter hormaechei

**Figure 4:**
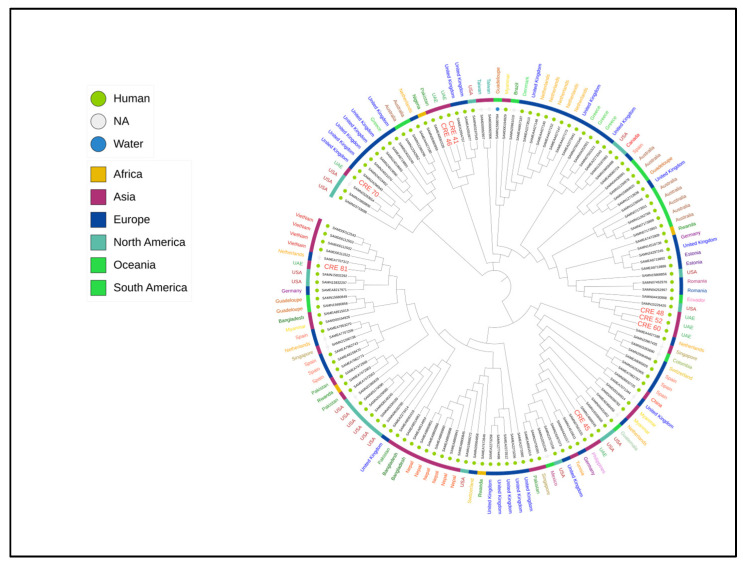
Circular visualization of k-mer sequence comparisons (approximately 4.7–5.3 megabases) of the eight *Enterobacter hormaechei* isolates investigated in this study with 125 publicly available *Enterobacter hormaechei* genomes. The maximum-likelihood tree in the Figure describes the SNP differences. From the inner to the outer circle: the inner circle indicates publicly available genomes of *Enterobacter hormaechei*, with the names from the current UAE study marked in red and in a larger font size; the second circle shows the host from which the *Enterobacter hormaechei* was isolated; the third circle indicates the country of isolation.

Phylogenetic analysis based on k-mer analysis of publicly available and current *Enterobacter hormaechei* isolates revealed that the isolates from the UAE did not converge into a single, homogeneous clonal group. Instead, these isolates were genetically diverse, aligning with several distinct genotypic clusters previously identified in *Enterobacter hormaechei*. These clusters encompass a range of isolates from different geographic regions worldwide, including both clinical and environmental sources. This diversity, as illustrated in Figure 4, highlights the complex genetic landscape of *Enterobacter hormaechei*, which has implications for epidemiological surveillance and infection control strategies. The presence of diverse genotypic clusters within the UAE isolates suggests multiple introduction pathways and broad dissemination of different genetic variants across global populations.

## 3. Discussion

*Enterobacter hormaechei*, first characterized and named in 1989, has gained prominence as a significant pathogen within healthcare settings [14]. Its distinctive characteristic lies in its capability to survive in the hospital environment, thereby developing resistance to multiple antibiotics and acting as a reservoir for infection and the transmission of drug resistance in nosocomial infections [15,16]. In a formal context, certain rare strains of *Enterobacter hormaechei* have been found to carry ESBLs and have increased production of AmpC cephalosporinase [17]. Subsequently, there have been reports of *Enterobacter hormaechei* strains producing carbapenemases worldwide, as shown in our present study and confirmed by other studies [18,19,20].

Several genes associated with antibiotic resistance were detected in our collection, contributing to the observed extensive resistance profile of the isolates. The combined action of different carbapenemase classes is likely responsible for their increased resistance to carbapenems and other antibacterial agents [21]. In particular, there have been reports that Enterobacterales co-host several carbapenemase determinants [22,23,24]. While recent evidence has shown that *Enterobacter hormaechei* ST93 simultaneously harbors *bla*_NDM-1_ and *bla*_KPC-2_ [20], our study is the first to detect the epidemic *Enterobacter hormaechei* ST171 (CRE81) clone that simultaneously harbors *bla*_NDM-1_ and *bla*_OXA-48_. This international high-risk clone, previously identified as a propagator of clinically important resistance genes in various international studies [12,25,26], was not observed as a dual carbapenemase producer, as documented in our current study. This finding highlights the increased pathogenicity of this bacterium, contributing to its MDR capability and potential to cause serious infections.

Rare carbapenemase genes have been documented in *Enterobacter hormaechei* in various studies, such as *bla*_IMP-1_ associated with ST89 and ST1103 lineages [19,20] and *bla*_VIM-2_ in *Enterobacter hormaechei* ST90 [27]. It is worth noting that *bla*_VIM-4_ has previously only been reported in other *Enterobacteriaceae* isolates from Kuwait and the United Arab Emirates [28,29]; however, our study revealed the presence of *bla*_VIM-4_ in an *Enterobacter hormaechei* ST269 (CRE70) isolate, which is partially consistent with a previous report of *bla*_VIM-4_ in *Enterobacter hormaechei*, although it was associated with another lineage (specifically, ST133 in Egypt). These results collectively suggest the widespread distribution and accumulation of the *bla*_VIM-4_ carbapenemase gene across different lineages of this bacterium [30].

Carbapenemase-producing *Enterobacter hormaechei* strains have been documented to exhibit resistance to alternative antibiotics, particularly colistin and tigecycline, which are often used as a last resort instead of carbapenems [31]. For example, colistin has been reintroduced into clinical practice as a therapeutic option to treat these severe infections [32]. Unfortunately, the global spread of plasmid-mediated colistin resistance genes (*mcr*) has posed tremendous challenges with regard to the clinical efficacy of colistin [33]. In our present study, five isolates (CRE41, CRE46, CRE48, CRE52, and CRE60) from two different lineages (ST124 and ST90) were observed to exhibit phenotypic resistance to colistin. Interestingly, the lack of *mcr* genes in all isolates suggests an alternative, possibly chromosomally mediated mechanism responsible for the observed resistance. This aligns with the roles of the two-component systems (TCSs) PmrA–PmrB (PmrAB) and PhoP–PhoQ (PhoPQ) in regulating colistin resistance. The PmrA–PmrB system, controlled by the pmrCAB operon and activated by PhoP–PhoQ, manages lipid A modification in Gram-negative bacteria. Under conditions of low magnesium or exposure to sub-lethal levels of cationic antimicrobial peptides such as polymyxin, these TCSs become activated. Chromosomal mutations in TCSs have been linked to colistin resistance in various Gram-negative bacteria, including *K. pneumoniae*, *P. aeruginosa*, *Salmonella* spp., *A. baumannii*, *E. coli*, and *Enterobacter* spp. This indicates that the observed colistin resistance in our isolates may similarly be mediated through chromosomal mutations affecting these TCSs or other mechanisms that have not yet been clearly explored in *Enterobacter* species [9].

A clonal outbreak involving the presence of *bla*_NDM-1_-producing *Enterobacter hormaechei* has been documented in China, attributed to the international high-risk clone ST78 [34]. In parallel, several secondary outbreaks characterized by NDM-producing *Enterobacter hormaechei*, with different lineages including ST89, ST146, ST198, and ST1303, occurred in Poland [35]. Through our own research, we identified the association of *bla*_NDM-1_ with two distinct lineages, specifically ST124 (CRE41 and CRE46) and ST90 (CRE48, CRE52, and CRE60). Notably, both lineages showed phenotypic resistance to colistin. It is noteworthy that the ST90 lineage was significantly predominant in our collection and was exclusively associated with the presence of *bla*_NDM-1_, a trend that mirrors previous findings in a Romanian isolate [29]. However, it is important to highlight that, in other studies, the *Enterobacter hormaechei* ST90 lineage has shown associations with different carbapenemase genes, namely *bla*_OXA-436_ in Denmark, *bla*_IMP-4_ in Australia, and *bla*_KPC-2_ in Canada [36].

All isolates possessed *ampC* β-lactamase genes, predominantly *bla*_ACT_ genes, indicating a conserved presence of inducible ACT-AmpC enzymes among the ECC members. Interestingly, species-specific patterns were observed in ACT-type β-lactamase genes; for example, *bla*_ACT-2_ and *bla*_ACT-3_ were found exclusively in *Enterobacter asburiae*, while *bla*_ACT-9_, *bla*_ACT-12_, and *bla*_ACT-6_ were only found in *Enterobacter kobei*, *Enterobacter ludwigii*, and *Enterobacter mori*, respectively [3]. In the case of our isolates belonging to *Enterobacter hormachei*, *bla*_ACT-7_, *bla*_ACT-15_, and *bla*_ACT-16_ were identified.

Only limited data are available on the global distribution of ST182. A Greek study identified this clone in association with *bla*_NDM-1_ [37], while, in our study, it was found to be linked with *bla*_OXA-48_, although this association was only observed in one isolate (CRE45).

Our results delineate the genomic context of the *bla*_NDM-1_ gene, surrounded by a pattern of resistance genes and mobile genetic elements, similar to findings in other studies that highlight the role of mobile elements in the dissemination of *bla*_NDM-1_ across different bacterial hosts. Notably, genes such as *aph(3′)-VI*, which is associated with aminoglycoside resistance, and elements such as IS30, which has been frequently identified in carbapenem-resistant isolates, suggest a robust mechanism for the persistence and spread of resistance. The presence of additional resistance determinants such as the bleomycin resistance gene (ble), isomerase, and reductase further complicate the resistance phenotype. Furthermore, we observed the *bla*_OXA-48_ gene to be consistently associated with the LysR transcriptional regulator, which has been documented to play a role in enhancing gene expression and resistance spread. The observed discrepancy between the susceptibility of CRE-45 to imipenem (resistant) and meropenem (susceptible) can be explained by the specific activity of OXA-48 enzymes. OXA-48 enzymes are carbapenemases, or more specifically, imipenemases with weak turnover rates for other carbapenems such as meropenem and ertapenem. This means that while CRE-45 exhibits resistance to imipenem due to the efficient hydrolysis by OXA-48, the enzyme’s weaker activity against meropenem results in a lower resistance level, allowing for the isolate to be susceptible or intermediate to meropenem. Similarly, the co-location of *bla*_VIM-4_ with aminoglycoside and trimethoprim resistance genes in CRE70 highlights the accumulation of resistance genes that enable survival under antimicrobial pressure.

The type VI secretion system (T6SS) plays a crucial role in bacterial virulence by aiding colonization under competitive conditions, contributing to pathogenesis through macrophage survival and biofilm formation, and killing neighboring non-immune bacteria by injecting antibacterial proteins. This enhances the bacteria’s ability to compete for resources. T6SS is also linked to virulence against eukaryotic host cells, posing a significant threat to human health. In our study, we observed that all isolates possessed this secretion system, consistent with the presence of T6SS. This system may explain the extensive drug resistance and virulence seen in our *Enterobacter hormaechei* isolates. Additionally, adhesins such as type 3 fimbriae and curli fibers, which were variably present among our isolates, facilitate cell adhesion, host cell invasion, and interaction with the host immune system, contributing to inflammatory responses. This complex interplay of virulence factors highlights the multifaceted pathogenic strategies employed by *Enterobacter hormaechei*, complicating treatment and control efforts [38].

*Enterobacter hormaechei* appears to have a selective advantage in a particular environment, and their ability to adapt to the hospital is further enhanced through the accumulation of numerous mobile genetic elements, including resistance and virulence genes. These pathogens often exhibit an MDR phenotype, as shown in the current study, which further reinforces their epidemic behavior. Due to these cumulative features, outbreak strains are likely to exhibit an antibiotic-resistant phenotype, thus making treatment difficult.

Accurate identification of ECC species is critical and highlights the importance of studying carbapenem-resistant genes within ECC. Equally important is the study of the mechanisms of horizontal gene transfer (HGT) in the gene’s vicinity. The presence of ECC with dual carbapenemase genes indicates increased bacterial selection pressure, requiring effective monitoring of their occurrence in clinical settings.

Although these pathogens are part of the ESKAPE group, they remain underestimated and understudied. Their presence in hospital settings, while not widespread, positions them as significant reservoirs of antimicrobial resistance. They facilitate the spread of resistance genes through HGT, further complicating infection control efforts and antimicrobial stewardship. Our study, while comprehensive, has certain limitations, including a limited sample size and lack of experimental validation for proposed resistance mechanisms, such as chromosomal mutations, efflux pumps, porin mutations, or other mechanisms involved in colistin resistance. Additionally, misidentification of *Enterobacter hormaechei* in hospital settings complicates accurate diagnosis and treatment. Clinically, our findings highlight the need for robust infection control measures to prevent the spread of dual carbapenemase-producing *Enterobacter hormaechei* strains, which exhibit resistance to multiple last-resort antibiotics, complicating treatment options and emphasizing the importance of routine surveillance and molecular diagnostics. Future research should address these limitations by including larger, diverse sample sizes and detailed genomic analyses to uncover the full spectrum of resistance mechanisms. Experimental validation of resistance pathways, including chromosomal mutations, efflux pumps, porin mutations, and other mechanisms, as well as investigating alternative therapeutic strategies, such as combination therapy or novel antimicrobial agents, is crucial.

## 4. Materials and Methods

### 4.1. Strain Collection

A total of eight non-repeat carbapenem-resistant *Enterobacter* strains were tested in our study, drawn from a larger collection of CRE samples collected between 2017 and 2019. They were isolated from different patients in two hospitals (A and B) in the Emirate of Abu Dhabi and from various infection sites, including wound swabs, sputum, tissue, and urine. These bacterial isolates were submitted to the Department of Medical Microbiology and Immunology, UAE University. Strains were stored at −80 °C in Tryptic Soy Broth (MAST, Merseyside, UK) with 20% glycerol. These specific eight strains were chosen for further characterization and analysis based on their WGS results, which revealed they belonged to *Enterobacter hormaechei*, a unique finding given that some of them were initially misidentified in the hospital and were received as belonging to other species.

### 4.2. Antibiotic Susceptibility Testing

The antibiotic susceptibility test aimed to assess the susceptibility of the isolates to different antibiotics. The isolates were tested using the Vitek2 compact system (Biomerieux, Craponne, France). The AST-N419 card was used to evaluate the following antibiotics: ampicillin/sulbactam, cefepime, ceftazidime, cefotaxime, ceftazidime/avibactam, ceftolozane/tazobactam, piperacillin/tazobactam, imipenem, meropenem, gentamicin, amikacin, ciprofloxacin, tigecycline, and co-trimoxazole.

The MIC of colistin was determined through broth microdilution (BMD) in cation-adjusted Muller–Hinton broth (Oxoid, Basingstoke, UK), using colistin sulfate (Sigma–Aldrich, St. Louis, MO, USA). Quality control was ensured using the *E. coli* ATCC 25922 strain. The interpretation of the susceptibility testing results followed the recommendations of the Clinical Laboratory Standards Institute (CLSI), as stated in the CLSI guidelines [39]. However, for tigecycline, the interpretation followed the European Committee on Antimicrobial Susceptibility Testing (EUCAST) guidelines [40]. Strains were categorized as multi-drug resistant (MDR) if they exhibited non-susceptibility to three or more different classes of antibiotics among those tested.

### 4.3. Whole-Genome Sequencing

Total genomic DNA extraction from the isolates was performed using the commercial Wizard^®^ Genomic DNA Purification Kit (Promega, Madison, WI, USA) according to the manufacturer’s instructions. WGS was then performed using the Illumina NovaSeq platform, generating paired-end reads of 150 base pairs. Genome assemblies were generated from the sequencing reads of the isolates using Unicycler v0.48 with default parameters [41]. Quality control of assemblies was assessed based on quast v5.2.0 [42].

### 4.4. Resistance Gene Content, Virulence Genes, and Plasmid Analysis

To determine resistance gene content, ResFinder 4.1 was used with default parameters [43,44]. Virulence genes were detected using VDFB (available at http://www.mgc.ac.cn/VFs/, accessed on 14 February 2024). Plasmid replicon types were identified using PlasmidFinder version 2.1 [45], with an identity percentage threshold set higher than 95% and a coverage cutoff greater than 90%.

### 4.5. Plasmid Analysis and Genetic Environment

Plasmid analysis in the study was conducted using Ridom SeqSphere+, which includes the MOB-suite tool (version 3.1.4) [46]. This combination facilitates comprehensive plasmid characterization, which is crucial for understanding the spread of antibiotic resistance. MOB-suite helps to identify and classify plasmids from bacterial genomes, focusing on resistance genes and mobility elements, thereby aiding in the investigation of genetic mechanisms underlying antibiotic resistance. Genetic environment comparisons and visualizations were performed using Mummer2circos (https://github.com/metagenlab/mummer2circos, accessed on 18 March 2024) and pyGenomeViz (https://github.com/moshi4/pyGenomeViz, accessed on 21 March 2024).

### 4.6. Phylogenetic Tree

Public isolates with sequence types identical to those found in this study were obtained from Pathogenwatch (https://pathogen.watch/, accessed on 28 March 2024). A k-mer analysis was performed for isolates retrieved from Pathogenwatch and those in this study using kSNP v3.10 [47], with default parameters, a k-mer size of 19, and maximum likelihood tree generation. The generated tree was uploaded to iTOL [48].

## 5. Conclusions

Our study highlighted the significant pathogenicity and multidrug resistance (MDR) capabilities of *Enterobacter hormaechei*, especially within healthcare settings. The obtained isolates exhibited extensive resistance to antibiotics, including carbapenems and colistin, often mediated by genes such as *bla*_NDM-1_, *bla*_OXA-48_, and *bla*_VIM-4_. The detection of high-risk clones, such as ST171 carrying dual carbapenemase genes, highlights the potential for serious infections and cross-transmission within hospital environments. The absence of *mcr* genes in colistin-resistant isolates suggests alternative, possibly chromosomally mediated resistance mechanisms. The presence of the type VI secretion system (T6SS) and various adhesins further contributes to the virulence and competitive advantage of *Enterobacter hormaechei*. Our findings emphasize the need for rigorous surveillance, strategic antibiotic stewardship, and the implementation of hospital-based whole-genome sequencing (WGS) surveillance to manage and mitigate the spread of these highly resistant and virulent pathogens. Accurate identification and monitoring of *Enterobacter cloacae* complex (ECC) species and their resistance mechanisms are crucial for effective infection control and treatment strategies.

## Figures and Tables

**Table 1:** Characterization of *Enterobacter hormaechei* isolates and associated sequence types.

Isolate	Species	Hospital	Sample Type	Sequence Type (ST)
CRE-41	*Enterobacter hormaechei* subsp. *Steigerwaltii*	A	Wound swab	124
CRE-45	*Enterobacter hormaechei*	B	Sputum	182
CRE-46	*Enterobacter hormaechei* subsp. *steigerwaltii*	A	Tissue	124
CRE-48	*Enterobacter hormaechei*	A	Sputum	90
CRE-52	*Enterobacter hormaechei*	A	Sputum	90
CRE-60	*Enterobacter hormaechei*	A	Sputum	90
CRE-70	*Enterobacter hormaechei* subsp. *Hormaechei*	B	Sputum	269
CRE-81	*Enterobacter hormaechei* subsp. *Xiangfangensis*	B	Urine	171

**Table 2:** Minimum inhibitory concentration (MIC) results and antibiotic resistance profiles of *Enterobacter hormaechei* isolates.

Isolate	PTZ	CTX	CAZ	C/A	C/T	CPM	IMI	MEM	AK	GM	CIP	TIG	COL	TS
CRE-41	≥128	≥64	≥64	≥16	≥32	≥32	8	≥16	16	≥16	≥4	≤0.5	8	≤20
CRE-45	≥128	≥64	≥64	1	≥32	≥32	4	2	4	≥16	≥4	≥8	≤0.5	≥320
CRE-46	≥128	≥64	≥64	≥16	≥32	≥32	8	≥16	16	≥16	≥4	≤0.5	8	40
CRE-48	≥128	≥64	≥64	≥16	≥32	≥32	8	≥16	32	≥16	≥4	≤0.5	8	≥320
CRE-52	≥128	≥64	≥64	≥16	≥32	≥32	8	≥16	32	≥16	≥4	≤0.5	8	≥320
CRE-60	≥128	≥64	≥64	≥16	≥32	≥32	≥16	≥16	32	≥16	≥4	≤0.5	8	≥320
CRE-70	≥128	32	≥64	≥16	≥32	2	8	≥16	16	≥16	1	≤0.5	≤0.5	≥320
CRE-81	≥128	≥64	≥64	≥16	≥32	≥32	≥16	≥16	32	≥16	≥4	≤0.5	≤0.5	≤20

PTZ: piperacillin/tazobactam; CTX: cefotaxime; CAZ: ceftazidime; C/A: ceftazidime/avibactam; C/T: ceftolozane/tazobactam; CPM: cefepime; IMI: imipenem; MEM: meropenem; AK: amikacin; GM: gentamicin; CIP: ciprofloxacin; TIG: tigecycline; COL: colistin; TS: co-trimoxazole. Red denotes resistance, yellow signifies intermediate resistance, and green represents susceptibility.

**Table 3:** Virulence gene distribution in *Enterobacter hormaechei* isolates.

VF Class	Related Genes	CRE45	CRE41	CRE46	CRE48	CRE52	CRE60	CRE70	CRE81
		ST 182	ST 124	ST 124	ST 90	ST 90	ST 90	ST 269	ST 171
Adherence									
• Curli (operon csg)	*csgC*								
• P fimbriae	*papC*								
• Type I fimbriae	*fimA*, *fimC*, *fimD*								
• Type 3 fimbriae	*mrkA*, *mrkB*, *mrkD*								
Autotransporter	*ehaB*								
Invasion									
• Invasion of brain endothelial cells (Ibes)	*ibeB*								
• Flagella (Burkholderia)	*cheB*, *cheW*, *cheR*, *cheY*, *motA*								
Iron uptake									
• Aerobactin siderophore	*iucA*, *iucB*, *iucC*, *iucD*, *iutA*								
• Salmochelin siderophore	*iroB*, *iroC*, *iroD*, *iroE*, *iroN*								
• Yersiniabactin siderophore (irp)	*irp1*, *irp2*								
• Enterobactin siderophore	-								
Secretion system (T6SS)	-								
Toxin (Cytotoxin, Shiga-toxin)	-								
Hemolysin	*hlyA*, *hlyB*, *hlyC*, *hlyD*								
Biofilm formation	*adeG*, *pgaC*								
Virulence factor MviM	*MviM*								
Virulence factor VirK	*virK*								
Immune evasion	*gale*, *gtrA*								
Serum resistance	*-*								
Virulence protein MsgA	*msgA*								

Green indicates gene presence, while red indicates gene absence.

**Table 4:** Plasmid types and associations with resistance genes in *Enterobacter hormaechei* isolates.

Isolate	Plasmid Size (bp)	Resistance Genes	Rep Type(s)	Relaxase Type(s)	Predicted Mobility
CRE41	115,921	*bla*_NDM1_, *bla*_CTX-M-15_, *qnrS1*, *aadA1*	IncFII	MOBF	conjugative
CRE45	45,454	*bla*_OXA-48_	IncL/M	-	non-mobilizable
CRE46	116,411	*bla*_NDM1_, *bla*_CTX-M-15_, *qnrS1*, *aadA1*	IncFII	MOBF	conjugative
CRE48	149,926	*bla*_NDM1_, *rmtC*, *sul1*, *dfrA14*	IncFII	MOBF	conjugative
CRE52	150,660	*bla*_NDM1_, *rmtC*, *sul1*, *dfrA14*	IncFII	MOBF	conjugative
CRE60	151,476	*bla*_NDM1_, *rmtC*, *sul1*, *dfrA14*	IncFII	MOBF	conjugative
CRE70	172,757	*bla*_VIM-4_, *mphA*, *sul2*, *cmlA5*, *aac(6′)-II*	IncC	MOBH	conjugative
CRE81	95,149	*bla*_NDM-1_, *bla*_TEM-1_, *qnrS1*, *aadA1*	IncFII	MOBP	conjugative
	69,442	*bla*_OXA-48_, *bla*_OXA-1_, *dfrA14*	IncL/M	MOBP	conjugative

## Data Availability

The datasets presented in this study can be found in online repositories (PRJNA1105356). The names of the repository/repositories and accession number(s) can be found below: https://www.ncbi.nlm.nih.gov/sra/PRJNA1105356, accessed on 27 April 2024.
